# Comparison of the risks of occupational diseases, avoidable hospitalization, and all-cause deaths between firefighters and non-firefighters: A cohort study using national health insurance claims data

**DOI:** 10.3389/fpubh.2022.1070023

**Published:** 2023-01-16

**Authors:** Woo-Ri Lee, Haejong Lee, Eun Woo Nam, Jin-Won Noh, Jin-Ha Yoon, Ki-Bong Yoo

**Affiliations:** ^1^Division of Cancer Control and Policy, National Cancer Control Institute, National Cancer Center, Goyang-si, Republic of Korea; ^2^Division of Health Administration, College of Software and Digital Healthcare Convergence, Yonsei University, Wonju-si, Republic of Korea; ^3^Department of Preventive Medicine and Institute of Occupational Medicine, Yonsei University College of Medicine, Seoul, Republic of Korea

**Keywords:** occupational diseases, ambulatory care sensitivity condition, firefighter, NHI cohort data, propensity score matching, Cox proportional hazard model, average treatment effect on the treated (ATT), avoidable hospitalization

## Abstract

**Objectives:**

National Health Insurance claims data were used to compare the incidence of occupational diseases, avoidable hospitalization, and all-cause death standardized incidence ratio and hazard ratio between firefighters and non-firefighters.

**Methods:**

The observation period of the study was from 2006 to 2015 and a control group (general workers and national and regional government officers/public educational officers) and a firefighter group was established. The dependent variables were occupational diseases, avoidable hospitalization (AH), and all-cause death. The analysis was conducted in three stages. First, the standardized incidence ratios were calculated using the indirect standardization method to compare the prevalence of the disease between the groups (firefighter and non-firefighter groups). Second, propensity score matching was performed for each disease in the control group. Third, the Cox proportional hazards model was applied by matching the participants.

**Results:**

The standardized incidence ratio and Cox regression analyses revealed higher rates of noise-induced hearing loss, ischemic heart disease, asthma, chronic obstructive pulmonary disease, cancer, back pain, admission due to injury, mental illness, depression, and AH for firefighters than general workers. Similarly, the rates of noise-induced hearing loss, ischemic heart disease, asthma, chronic obstructive pulmonary disease, back pain, admission due to injury, mental illness, depression, and AH were higher in the firefighter group than in the national and regional government officer/public educational officer group.

**Conclusions:**

The standardized incidence ratios and hazard ratios for most diseases were high for firefighters. Therefore, besides the prevention and management of diseases from a preventive medical perspective, management programs, including social support and social prescriptions in the health aspect, are needed.

## 1. Introduction

Firefighters are responsible for various safety activities, such as fire prevention, fire response, 119 (Korean emergency care number) rescue/emergency calls, and 119 related life safety activities in South Korea. Over the last decade, the number of dispatches and rescues has annually increased by 8.5 and 9.7%, respectively. Compared to 10 years ago, the dispatch and rescue numbers in 2019 doubled to 115 and 136%, respectively. Thus, firefighters play a role in ensuring the safety of people through various activities (1). Firefighters are engaged in hazardous tasks, such as fire suppression, emergency, and rescue services. Owing to occupational characteristics, various harmful factors that threaten health, including chemical factors (e.g., harmful gases) and physical factors (e.g., noise and high temperature) may expose firefighters to severe physical and mental danger.

Firefighters with long working hours and shift work are exposed to tension for a long time. Owing to chronic stress and sleep disorders that subsequently develop, a decline in the physical and mental health and quality of life of firefighters occurs (2). The direct and indirect experiences of shocking, traumatic incidents, which are common owing to excessive working hours and the occurrence of repeated disasters, facilitate the development of post-traumatic stress disorders in firefighters (3). In a prior study, firefighters were reported to have greater degrees of hearing loss than the general population, and the degree of hearing loss was found to increase with age (4). Moreover, in the event of a fire, various harmful chemical factors, including carbon monoxide, carbon dioxide, hydrogen cyanide, and nitrogen oxides generated by furniture, insulating materials, plastics, etc., are present, which pose a health risk to firefighters who are exposed to these toxic gases for long and short periods (5–8).

Owing to various adverse health factors, firefighters are at risk of developing occupational diseases, such as noise-induced hearing loss (NIHL), cardiovascular disease, respiratory disease, musculoskeletal disease, occupational cancer, and mental illness. Furthermore, in a study on firefighters in a Busan metropolitan city, 22.8% of firefighters were found to have diabetes while 10.7% had hypertension. Accordingly, the health of firefighters was not ideal (9), especially for those with diabetes and hypertension, which are ambulatory care sensitive conditions (ACSCs) in which unnecessary hospitalization can be avoided through timely and effective outpatient care (10). The experience of avoidable hospitalization (AH) is closely related to the quality of primary care and medical accessibility (10, 11). For firefighters, continuous health management is necessary due to the nature of their work.

This study aimed to highlight the necessity of health management for firefighters by using the National Health Insurance (NHI) claims data from South Korea to compare the incidence of occupational diseases, AH, and all-cause deaths standardized incidence ratios (SIRs) and hazard ratios (HRs) between firefighters and non-firefighters.

## 2. Materials and methods

### 2.1. Data

The NHI claims data from South Korea were used as the research data. NHI claims data are data provided by the NHI and include qualifications and premiums for all populations, health examination results, personal medical histories, long-term care insurance data for older adults, the current status of hospitals, and registration information for cancer and rare diseases; national medical care data for 1.3 trillion cases are also included in this database (12).

A cohort of data was collected from 2005 to 2015. The year 2005 was used as the washout period to exclude illnesses. The 10-year data collected from 2006 to 2015 were used for the analysis. Firefighters were placed in one group, general workers formed a control group, and national and regional government officers (NRGs) and public educational officers (PEOs) known to have few occupational risk factors formed another control group. Accordingly, the study participants were divided into firefighters and general workers comparison group and the firefighters and NRGs/PEOs comparison group.

**Figure 1:**
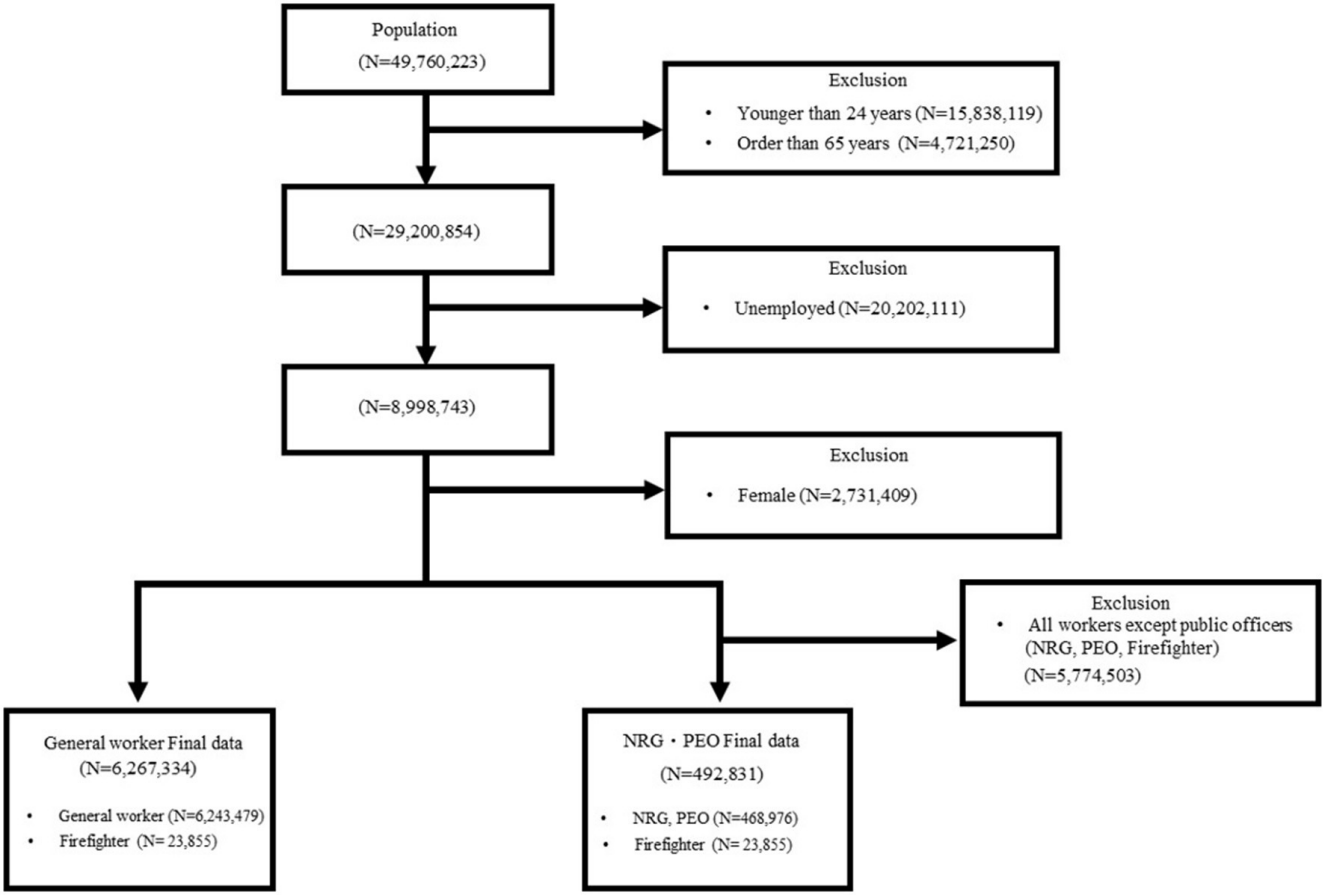
Process used to select the study population.

Of the 49,760,223 participants, only age group of 25–64 years in 2006 (when the observation started) were included in the study (*N* = 20,559,369 excluded). Further, those who were unemployed and female were excluded (*N* = 22,933,520 excluded). Additionally, men that had used medical services as part of a principal diagnosis for a disease corresponding to the dependent variable one year before the start of the observation period for each disease were excluded from the study. All exclusion criteria were the same for both groups, except that only public officers were studied in the NRG/PEO control group (*N* = 5,774,503 excluded). The final general worker group included 6,267,334 people between 25 and 64 years, while the NRG/PEO control group consisted of 492,831 people (Figure 1). This study was approved by the Institutional Review Board of Yonsei University (1041849-202203-SB-057-01).

### 2.2. Study variables

#### 2.2.1. Dependent variables

The dependent variables were occupational diseases, AH, and all-cause deaths. Only participants with principal diagnoses based on the International Classification of Diseases 10th revised edition (ICD-10) codes were included in the analyses of occupational diseases and AH.

The following occupational diseases were considered: NIHL (H83.3), ischemic heart disease (IHD) (I20–I25), myocardial infarction (I21–I22), cerebral infarction (I63), cerebral hemorrhage (I60–I62), asthma (J45 and J46), chronic obstructive pulmonary disease (COPD) (J43.1, J43.2, J43.8, J43.9, J44), cancer (C00–C97), back pain (M54), admission due to injury (S00–T98), mental illness (F00–F99), and depression (F32–F33). Based on disease prevalence, the participants were classified as “1” (occurred) and “0” (did not occur).

AH is an indicator closely related to the quality of primary care and accessibility to care, with the concept that unnecessary hospitalization can be avoided through proper management of primary care. Therefore, in this study, participants who were hospitalized for the relevant ACSCs in South Korea were classified as “1” and those who were not hospitalized were classified as “0.” The variables included those hospitalized (as a principal diagnosis) (13) with ICD-10 code-based meningococcal infections (A39); emphysema (J43); meningitis in bacterial diseases classified elsewhere (G01); bacterial meningitis, not elsewhere classified (NEC) (G00); nondiabetic hypoglycemic coma (E15); other disorders of fluid, electrolyte, and acid-base balance (E87); convulsions, NEC (R56); other disorders of pancreatic internal secretion (E16); volume depletion (E86); other acute IHDs (I24); other respiratory disorders (J98); pneumonia (J09–J18); unspecified diabetes mellitus (E14); fever of other and unknown origin (R50); dizziness and giddiness (R42); angina pectoris (I20); essential hypertension (I10); other noninfectious gastroenteritis and colitis (K52); influenza, virus not identified (J11); cutaneous abscess, furuncle, and carbuncle (L02); acute tonsillitis (J03); acute upper respiratory infections (J00, J01, J02, J04, and J05); acute upper respiratory infections of multiple and unspecified sites (J06); and chronic rhinitis, nasopharyngitis, and pharyngitis (J31).

Deaths from all causes were included. Participants who died during the observation period were classified as “1” and survivors were classified as “0.”

#### 2.2.2. Independent variable

The independent variable was the occupation type. To compose the data set, firefighters were coded as “1” and general workers and NRG/PEOs were coded as “0.”

#### 2.2.3. Control variables

The control variables were demographic and health-related. The demographic variables included age, income, and region, while the health-related variables included disability, Charlson comorbidity index (CCI) scores, and admission in 2005.

The demographic variable, age, was divided into eight 5-year categories: 25-29, 30-34, 35-39, 40-44, 45-49, 50-54, 55-59, and 60-64 years. Income was divided into four groups: NHI premiums of 70% or less (quartile 1), 80% or less (quartile 2), 90% or less (quartile 3), and 100% or less (quartile 4). The regions were divided into Seoul, Gyeonggi, metropolitan, and rural. Metropolitan areas, excluding Seoul and Gyeonggi, were classified as large cities, while other areas were classified as rural areas.

The presence or absence of a disability as a health-related variable was classified using the disability grade variable. The allocated disability grades ranged from 1 to 6; the lower the grade, the higher the severity. Grades 1–2 indicated severe disorders while grades 3–6 indicated mild disorders. In this study, participants with grades 1–6 were classified as “1,” and participants with no-disability were classified as “0” (14). The CCI score is a typical index for adjusting for the severity of comorbidities. There are several criteria for calculating the CCI score; however, Quan et al. (15) calculation method is typically used. Quan et al. (15) method selects 17 diseases and imposes weights according to the disease by scoring them from 0 to 6 points (15), with a minimum of 0 and a maximum of 24 points (15). In this study, the CCI score was divided into 0, 1, and 2 points or more. In the case of admission in 2005, admissions for all diseases were considered after applying the 1-year washout to the diseases corresponding to each dependent variable. The presence or absence of admission for illness was scored as “1” if admission occurred in 2005 and as “0” if admission did not occur in 2005, considering only the principal diagnosis.

### 2.3. Statistical analysis

The analysis was conducted in three stages. First, the SIR was calculated using the indirect standardization method to compare disease prevalence between the firefighter and control groups. The expected number of cases in the firefighter group was calculated based on the incidence rate by age in the control group. The SIR was calculated from the ratio between the actual and expected numbers of cases. The formula for calculating SIR is as follows:


$$\begin{array}{l} \;SIR=\frac{observed\;number\;of\;cases}{expected\;number\;of\;cases}\times 100\;(formula)\\ (where\;expected=person\;-year\;of\;case\;group\;\times \\ \;incidence\;rate\;of\;control\;group) \end{array}$$

The 95% confidence interval (CI) of the SIR was calculated assuming a Poisson distribution and was judged to be statistically significant when the 95% CI crossed 1. Second, propensity score matching (PSM) was performed for each disease in the control group. The propensity score was calculated using the variables of age, region, income, disability, CCI, and admission in 2005, and 1:3 matching was performed using the greedy matching method. Matching verification was verified by ensuring that the absolute value of the standardized difference was <0.1 (16). Finally, regression analysis using the Cox proportional hazard model was performed by matching the participants. Proportional risk assumptions were confirmed using a Kaplan–Meier survival curve (17), and the model was adjusted for age, region, income, disability, CCI, and admission in 2005. All analyses were performed using SAS version 9.4 (SAS Institute, Cary, NC, USA). The statistical significance of the hypothesis test was confirmed using a two-sided test.

## 3. Results

Age, income, region, disability, CCI score, and frequency of admission in 2005 were analyzed by group (Table 1). Based on age, 30.4% of the firefighters were 35–39 years old. Further, approximately 83% of firefighters had income in quartile 2 or above, indicating that most had a high income. Most firefighters lived in rural regions, were non-disabled, had CCI scores of 0, and had never been hospitalized in 2005. Most general workers were 35–39 years (19.0%) and had income in quartile 1 (58.3%). The residence, presence or absence of disability, CCI scores, and proportion of admissions in 2005 of general workers were similar to those of firefighters. Finally, 21.1% of the participants in the NRG/PEO group were 45–49 years old, and 80.8% had income in quartile 2 or above. Thus, most of these workers had a high income, similar to firefighters. Other variables for the NRG/PEO participants were found to be similar to those of firefighters.

**Table 1:** Characteristics of the general participants.

**Variable**	**Firefighter (*N =* 23,855)**	**Control group**	
		**General workers** ** (*N =* 6,243,479)**	**NRGs, PEOs (*N =* 468,976)**
	**(*N*, %)**	**(*N*, %)**	**(*N*, %)**
**Age**			
25–29	1,464 (6.1)	858,364 (13.7)	22,837 (4.9)
30–34	4,487 (18.8)	1,184,656 (19.0)	49,623 (10.6)
35–39	7,242 (30.4)	1,188,097 (19.0)	69,644 (14.9)
40–44	4,940 (20.7)	972,827 (15.6)	83,752 (17.9)
45–49	2,970 (12.5)	870,980 (14.0)	98,931 (21.1)
50–54	2,004 (8.4)	597,951 (9.6)	77,939 (16.6)
55–59	713 (3.0)	363,265 (5.8)	47,768 (10.2)
60–64	35 (0.1)	207,339 (3.3)	18,482 (3.9)
**Income**			
Quartile 1	4,066 (17.0)	3,639,709 (58.3)	90,036 (19.2)
Quartile 2	6,317 (26.5)	813,219 (13.0)	73,783 (15.7)
Quartile 3	11,143 (46.7)	857,267 (13.7)	145,197 (31.0)
Quartile 4	2,329 (9.8)	933,284 (14.9)	159,960 (34.1)
**Region**			
Seoul	2,929 (12.3)	1,332,494 (21.3)	87,157 (18.6)
Gyeonggi	5,118 (21.5)	1,521,675 (24.4)	84,748 (18.1)
Metropolitan	6,540 (27.4)	1,608,008 (25.8)	123,956 (26.4)
Rural	9,268 (38.9)	1,781,302 (28.5)	173,115 (36.9)
**Disability**			
Non-disabled	23,475 (98.4)	6,051,692 (96.9)	456,199 (97.3)
disabled	380 (1.6)	191,787 (3.1)	12,777 (2.7)
**CCI**			
0	20,829 (87.3)	5,531,753 (88.6)	403,282 (86.0)
1	1,749 (7.3)	384,196 (6.2)	34,070 (7.3)
≥2	1,277 (5.4)	327,530 (5.2)	31,624 (6.7)
**Admission in 2005**			
No	22,323 (93.6)	5,913,171 (94.7)	442,188 (94.3)
Yes	1,532 (6.4)	330,308 (5.3)	26,788 (5.7)

NRG, national and regional government officer; PEO, public education officer; CCI, Charlson Comorbidity Index.

We determined the frequency of occupational diseases, avoidable hospitalizations, and all-cause deaths in the population (Table 2). The total number of firefighters was 23,855. Based on disease, 89 had NIHL, 1,464 had IHD, 157 had myocardial infarction, 320 had cerebral infarction, 112 had cerebral hemorrhage, 2,745 had asthma, 919 had cancer, 9,791 had back pain, 4,479 were admitted due to injury, 3,078 had mental illnesses, and 857 had depression; a total of 1,169 avoidable hospitalizations and 251 all-cause deaths were recorded. Notably, for firefighters, general workers, and NRG/PEOs, back pain had the highest number of cases, followed by admission due to injury and mental illness.

**Table 2:** Disease and person-year data of firefighters, general workers, national regional government officers, and public educational officers.

**Number**	**Disease**	**Firefighter**		**Control group**			
				**General workers**		**NRGs, PEOs**	
		**Cases**	**PY**	**Cases**	**PY**	**Cases**	**PY**
1	NIHL	89	236,441	11,192	61,105,178	812	4,635,670
2	IHD	1,464	226,707	351,941	58,662,879	37,072	4,362,700
3	Myocardial infarction	157	236,025	47,005	60,882,490	4,207	4,613,857
4	Cerebral infarction	320	234,820	108,893	60,454,972	10,161	4,568,580
5	Cerebral hemorrhage	112	236,325	33,933	60,984,961	2,743	4,624,136
6	Asthma	2,725	215,129	657,276	56,115,920	53,344	4,214,524
7	COPD	446	234,120	127,193	60,351,753	11,641	4,563,050
8	Cancer	919	231,728	250,137	59,860,270	26,259	4,485,518
9	Back pain	9,791	163,993	2,191,515	45,770,680	165,913	3,447,576
10	Admission due to injury	4,479	210,593	756,235	56,916,312	47,610	4,372,331
11	Mental illness	3,078	212,454	693,428	56,100,241	61,252	4,156,905
12	Depression	857	230,659	174,393	59,929,016	16,847	4,508,904
13	AH	1,169	230,800	311,862	59,536,633	25,299	4,504,079
14	All cause deaths	251	237,007	113,225	61,179,099	7,417	4,641,439

PY, person-year; NIHL, noise-induced hearing loss; IHD, ischemic heart disease; COPD, chronic obstructive pulmonary disease; AH, avoidable hospitalization; NRG, national and regional government officer; PEO, public educational officer.

The SIRs for occupational diseases and AH, and the standardized mortality ratios (SMRs) for all-cause deaths for firefighters were compared to those of non-firefighters (Table 3). Compared to general workers, firefighters had higher rates of NIHL [2.09 (95% CI, 1.68–2.58)], IHD [1.21 (95% CI, 1.15–1.27)], asthma [1.13 (95% CI, 1.09–1.17)], COPD [1.16 (95% CI, 1.05–1.27)], cancer [1.15 (95% CI, 1.08–1.23)], back pain [1.28 (95% CI, 1.25–1.30)], admission due to injury [1.62 (95% CI, 1.58–1.67)], mental illness [1.23 (95% CI, 1.19–1.28)], depression [1.35 (95% CI, 1.26–1.44)], and AH [1.09 (95% CI, 1.03–1.15)]. In the case of death, the SMR of firefighters was lower than that of general workers [0.73 (95% CI, 0.65–0.83)]. Firefighters also had higher rates of NIHL [2.25 (95% CI, 1.81–2.77)], IHD [1.11 (95% CI, 1.05–1.17)], asthma [1.07 (95% CI, 1.03–1.11)], COPD [1.18 (95% CI, 1.07–1.29)], back pain [1.31 (95% CI, 1.29–1.34)], admission due to injury [1.92 (95% CI, 1.87–1.98)], mental illness [1.10 (95% CI, 1.06–1.14)], depression [1.08 (95% CI, 1.01–1.16)], and AH [1.16 (95% CI, 1.09–1.23)] than NRG/PEOs. However, regarding death, a statistical significance could not be confirmed between these groups.

**Table 3:** Standardized incidence ratios of firefighters compared to general workers, national regional government officers, and public educational officers.

**Number**	**Disease**	**General workers**		**NRGs, PEOs**	
		**SIR**	**95% CI**	**SIR**	**95% CI**
1	NIHL	**2.09**	(1.68–2.58)	**2.25**	(1.81–2.77)
2	IHD	**1.21**	(1.15–1.27)	**1.11**	(1.05–1.17)
3	Myocardial infarction	0.98	(0.83–1.15)	1.12	(0.95–1.31)
4	Cerebral infarction	1.00	(0.89–1.11)	1.08	(0.96–1.20)
5	Cerebral hemorrhage	0.94	(0.77–1.13)	1.05	(0.87–1.27)
6	Asthma	**1.13**	(1.09–1.17)	**1.07**	(1.03–1.11)
7	COPD	**1.16**	(1.05–1.27)	**1.18**	(1.07–1.29)
8	Cancer	**1.15**	(1.08–1.23)	1.06	(0.99–1.13)
9	Back pain	**1.28**	(1.25–1.30)	**1.31**	(1.29–1.34)
10	Admission due to injury	**1.62**	(1.58–1.67)	**1.92**	(1.87–1.98)
11	Mental illness	**1.23**	(1.19–1.28)	**1.10**	(1.06–1.14)
12	Depression	**1.35**	(1.26–1.44)	**1.08**	(1.01–1.16)
13	AH	**1.09**	(1.03–1.15)	**1.16**	(1.09–1.23)
14	All cause deaths	0.73	(0.65–0.83)	1.13	(0.99–1.28)

NRG, national and regional government officer; PEO, public educational officer; SIR, standardized incidence ratio; CI, confidence interval; NIHL, noise-induced hearing loss; IHD, ischemic heart disease; COPD, chronic obstructive pulmonary disease; AH, avoidable hospitalization. The bold values indicate the statistically significant values.

After PSM, the HRs of the occupational diseases, AH, and all-cause deaths for firefighters were compared with those of non-firefighters using regression analysis and the Cox proportional hazard model (Table 4). Firefighters were found to have a higher risk of NIHL [2.15 (95% CI, 1.64–2.82)], IHD [1.16 (95% CI, 1.10–1.24)], asthma [1.08 (95% CI, 1.03–1.12)], COPD [1.13 (95% CI, 1.01–1.26)], back pain [1.21 (95% CI, 1.18–1.24)], admission due to injury [1.74 (95% CI, 1.67–1.80)], mental illness [1.22 (95% CI, 1.17–1.27)], depression [1.34 (95% CI, 1.24–1.45)], and AH [1.09 (95% CI, 1.02–1.17)] than general workers. Compared to NRG/PEOs, firefighters also had a higher risk of NIHL [2.59 (95% CI, 1.95–3.44)], IHD [1.11 (95% CI, 1.04–1.17)], back pain [1.28 (95% CI, 1.25–1.31)], admission due to injury [1.86 (95% CI, 1.79–1.93)], mental illness [1.11 (95% CI, 1.06–1.16)], and AH [1.16 (95% CI, 1.08–1.24)]. Regarding the HR for death, statistical significance could not be confirmed.

**Table 4:** Hazard ratios and 95% confidence intervals for the risk of death from all causes and risk of diseases for firefighters compared to general workers, national regional government officers, and public educational officers after propensity score matching.

**Number**	Disease^*^	**General workers**		**NRGs, PEOs**	
		**HR**	**95% CI**	**HR**	**95% CI**
1	NIHL	**2.15**	(1.64–2.82)	**2.59**	(1.95–3.44)
2	IHD	**1.16**	(1.10–1.24)	**1.11**	(1.04–1.17)
3	Myocardial infarction	0.99	(0.83–1.19)	1.17	(0.97–1.40)
4	Cerebral infarction	0.98	(0.86–1.11)	1.08	(0.95–1.23)
5	Cerebral hemorrhage	1.09	(0.88–1.35)	1.09	(0.87–1.35)
6	Asthma	**1.08**	(1.03–1.12)	1.01	(0.96–1.05)
7	COPD	**1.13**	(1.01–1.26)	1.11	(0.99–1.24)
8	Cancer	1.06	(0.99–1.15)	1.06	(0.99–1.15)
9	Back pain	**1.21**	(1.18–1.24)	**1.28**	(1.25–1.31)
10	Admission due to injury	**1.74**	(1.67–1.80)	**1.86**	(1.79–1.93)
11	Mental illness	**1.22**	(1.17–1.27)	**1.11**	(1.06–1.16)
12	Depression	**1.34**	(1.24–1.45)	1.07	(0.99–1.16)
13	AH	**1.09**	(1.02–1.17)	**1.16**	(1.08–1.24)
14	All cause deaths	1.01	(0.88–1.17)	1.10	(0.95–1.27)

* Adjusted for Age, Income, Region, Disability, CCI, Admission in 2005; HR, hazard ratio; CI, confidence interval; NIHL, noise-induced hearing loss; IHD, Ischemic heart disease; COPD, chronic obstructive pulmonary disease; AH, avoidable hospitalization. The bold values indicate the statistically significant values.

## 4. Discussion

### 4.1. Key findings

This study aimed to compare the SIRs, SMRs, and risks of occupational diseases, AH, and all-cause deaths between firefighters and non-firefighters. Based on the key findings of this study, the SIRs and risks of NIHL, IHD, asthma, COPD, cancer, back pain, admission due to injury, mental illness, depression, and AH were higher for firefighters than non-firefighters.

### 4.2. Interpretation

Firefighters had a higher risk of NIHL than non-firefighters. These results were similar to those of a previous study that revealed more significant hearing loss in firefighters than in the general population and a higher probability of experiencing NIHL (4). Firefighters are often exposed to sirens, air horns, and engine noise. According to previous studies, wearing personal protective equipment (PPE) was associated with reduced incidence of NIHL (18) and stated that wearing PPE was necessary to reduce noise exposure (19). In the case of IHD, firefighters had a higher risk of developing cardiovascular disease than general workers; this result is consistent with that of a previous study (20, 21). Firefighters are at increased risk of developing cardiovascular disease due to frequent chemical exposures and inflammatory reactions caused by inhaled toxins (22). In addition, Various psychological stressors and shift work are also known to contribute to cardiovascular disease (23). Firefighters sometimes do not wear PPE in the presence of harmful inhalants after fire suppression, which contributes to their risk of developing cardiovascular disease (20).

Our results regarding lung disease, such as asthma and COPD, were similar to the following previous studies. The firefighters' standardized admission ratio due to asthma and COPD was higher in the military control group (24). Firefighters had a higher degree of deterioration of lung function than general workers (25). The pulmonary function of firefighters is associated with many factors, including firefighting exposure level, smoke, use of respiratory PPE, and other health behaviors (24, 26, 27). In particular, inconsistent use of respiratory PPE and chronic respiratory diseases have been identified to have interactive effects that reduce one's health-related quality of life (28). Accordingly, strict guidelines for wearing PPE are required to prevent NIHL and respiratory and heart disease development.

Contrary to the prior (29, 30), the all-cancer SIRs of the firefighters in this study were higher than those of non-firefighters. In previous studies (29, 30), firefighters appeared to be the healthier group due to the healthy worker effect. One prior study, which observed firefighters for a long period from 1985 to 2009, confirmed that firefighters had a significantly higher all-cancer incidence (31). Using Australian data, firefighters showed significantly high overall risk of cancer with data year from 1976 to 2003 (32). However, recent studies reported that there were no statistically significant overall risks of cancer among firefighters (29, 33, 34). The systematic review study reported a high level of heterogeneity of articles (29). These results depend on the cohort design definitions and cohort observation periods applied. Cohort study designs are important in occupational studies as occupational changes may occur depending on the time of turnover or retirement, and job security may change depending on the period of occupational observation. These thing and the incubation period of cancers make it difficult to identify the true effects (35). Studies performed with the fixed cohort method, which considers employment security by occupation, and the dynamic cohort method are necessary. Moreover, a systematic cohort of firefighters must be established and additional research must be conducted.

Our results regarding back pain and admission due to injury were the same as those of a previous study (36), where firefighters had a higher risk of musculoskeletal disorders (lumbar sprain, lumbar disc herniation, shoulder soft tissue disease, among others) and admission due to injury than general public officers. Owing to the intense physical labor performed by firefighters, there is a high possibility of back pain recurrence or admission due to injury. Therefore, the results of this study may have been underestimated. A previous study on injuries in firefighters at fire sites found that overworking and increased tension were the leading causes of injury, accounting for 26% of the total injuries. Other significant causes of injuries included slipping, falling, among others. Sprain was found to account for more than 28% of all injuries, and one-third of such trauma leads to loss of working hours (37). If the work burden of firefighters is increased in such situation, the health of the entire firefighting organization may be threatened.

In the case of mental illness, the firefighters in our study had the same results as those obtained in a previous study, where the risks of mental illnesses and mood disorders in firefighters were higher than those in general public officers (36). For firefighters, stress due to job instability, improper compensation, work culture, among others is significantly associated with depressive symptoms (38). Depression plays a significant role in the relationship between job stress and suicide committed by firefighters (39). Accordingly, the mental illnesses of firefighters should be managed and their work stress should be reduced. The work stress of firefighters is known to affect their quality of life due to a lack of social support (40). Thus, the mental health and stress of firefighters must be managed using social support systems.

AH is an indicator of the accessibility and quality of primary care (10, 11). Our study results are not the results of comparisons by region; thus, it is somewhat difficult to interpret the results according to the geographical accessibility of primary care and the quality of primary care. Therefore, the results can be interpreted as the accessibility level of medical use at the personal level. As a result, the shiftwork system must be reorganized to improve the accessibility of firefighters.

In the case of all-cause death, the SMR of the firefighter group was lower than that of the general worker's control group. When confirming the HR of all-cause death using Cox regression analysis, statistical significance could not be confirmed in any of the results when general workers and NRG/PEOs were employed as controls. The lower risk of all-cause death observed in firefighters compared to general workers might be due to the healthy worker effect resulting from the characteristics of the firefighters' occupation. These results were similar to those of a previous study, in which the SMR of firefighters was lower than that of the general population (41).

### 4.3. Strengths and limitations

This study had some limitations. First, using 2006 as the reference point, we determined the effect of time flow on the outbreak of illness; however, in the case of occupations that were independent variables, we could not consider changes due to period flow and occupational maintenance periods. In the case of public officers, job security differs between temporary public officers and NRG/PEOs, whose regular retirement is fixed. Consequently, dangerous effects may be mixed in the civil service group. However, efforts were made to minimize bias by applying PSM and controlling for covariates in the firefighter and control groups (16). Second, the effects of exposure associated with harmful substances affecting the health of firefighters were not considered. Special health examination data are required to understand the effects of exposure to harmful substances (42); such data could not be obtained from the NHI claim data. Third, the impact of the firefighters' job series was not considered. Firefighters' job series are divided into fire suppression, first aid, rescue, and administration. For administrative and indoor positions, there is a relatively low possibility of exposure to harmful substances compared to other locations. Consequently, if the effects of the series are not distinguished, firefighters may have a mixed risk of health problems. To overcome this limitation, the present study was only performed with male firefighters. A total of 5,299 of the 56,639 firefighters in 2019 were female (9.3%), and most performed administrative or indoor jobs (43). Accordingly, we included only male firefighters in this study and sought to control for the effects of series and sex imbalances. Fourth, Lifestyle-related factors are influential factors that are closely related to individual health, and the occurrence of diseases. However, this study did not control for lifestyle factors, such as smoking, drinking, and exercise. To overcome this limitation, health-related variables were used as surrogate indicators. Efforts were made to reflect the participants' health statuses using the variables of disability, CCI score, and admission in 2005.

Despite these limitations, our study provided a general description of the occupational diseases of firefighters and confirmed the risk of AH as a health management index. Further, the average treatment effect on the treated was estimated using the PSM method, thereby increasing the relevance of the results.

## 5. Conclusion

According to the study results, the lower risk of all-cause death observed in firefighters due to the healthy worker effect. However, the SIRs and HRs of firefighters were high for most diseases. Firefighters are always exposed to disaster risks and fatalities due to industrial disasters that occur every year. Therefore, besides prevention and management of diseases from a preventive medical perspective, management programs, such as social support and social prescriptions in the health aspect, are needed. It is hoped that such a management program will reduce the occurrence of occupational diseases among firefighters and the number of disabilities and deaths caused by such diseases. Therefore, it is necessary to conduct a study to evaluate the effectiveness of firefighters' preventive activities in the future.

## Data availability statement

Publicly available datasets were analyzed in this study. This data can be found at: https://nhiss.nhis.or.kr/bd/ab/bdaba000eng.do;jsessionid$=$B1rHeZOeazNhRerGsXWUUtdpyGklOnnx1LfuTqIrE1xZjiKQbzcZQIcasZAUuIPf.primrose2_servlet_engine10.

## Ethics statement

The studies involving human participants were reviewed and approved by the Institutional Review Board of Yonsei University (1041849-202203-SB-057-01). Written informed consent for participation was not required for this study in accordance with the national legislation and the institutional requirements.

## Author contributions

Conceptualization and methodology: W-RL, HL, EN, J-WN, J-HY, and K-BY. Formal analysis and writing–original draft: W-RL. Writing–review and editing: W-RL, J-HY, and K-BY. All authors contributed to the article and approved the submitted version.
